# 361. Evaluation of Resistance to Nirmatrelvir/ritonavir in Evaluation of Protease Inhibition for COVID-19 (EPIC) High-Risk and Standard-Risk Clinical Trials

**DOI:** 10.1093/ofid/ofad500.431

**Published:** 2023-11-27

**Authors:** Mary Lynn Baniecki, Shunjie Guan, Zhenyu Wang, Yan Chen, Weihang Bao, Wen He, Elizabeth Dushin, Craig Hyde, Yuao Zhu, Rhonda D Cardin, Jennifer Hammond, Sandeep Menon, Charlotte Allerton, Holly Soares

**Affiliations:** Pfizer Inc, Cambridge, Massachusetts; Pfizer, Cambridge, Massachusetts; Pfizer, Cambridge, Massachusetts; Pfizer, Inc, Medford, Massachusetts; Pfizer Inc, Cambridge, Massachusetts; Pfizer, Cambridge, Massachusetts; Pfizer, Cambridge, Massachusetts; Pfizer, Cambridge, Massachusetts; Pfizer, Inc., Pearl River, New York; Pfizer, Inc., Pearl River, New York; Pfizer Inc, Cambridge, Massachusetts; Pfizer, Cambridge, Massachusetts; Pfizer, Cambridge, Massachusetts; Pfizer, Cambridge, Massachusetts

## Abstract

**Background:**

Nirmatrelvir/ritonavir administered twice daily for 5 days (5D) (within 5 days of symptom onset) resulted in a clinically and statistically significant (6% absolute and 86% relative) risk reduction in COVID-19 related hospitalization or all cause death through Day 28 [1]. COVID-19 rebound regardless of treatment has been reported [2,3,4]. Here, an integrated analysis of EPIC-HR/SR [5,6] virology data was conducted to evaluate clinical resistance to nirm/r and if it is associated with viral load rebound (VLR) or progression to severe COVID-19 (i.e., hospitalization or death by D28).

**Figure:**
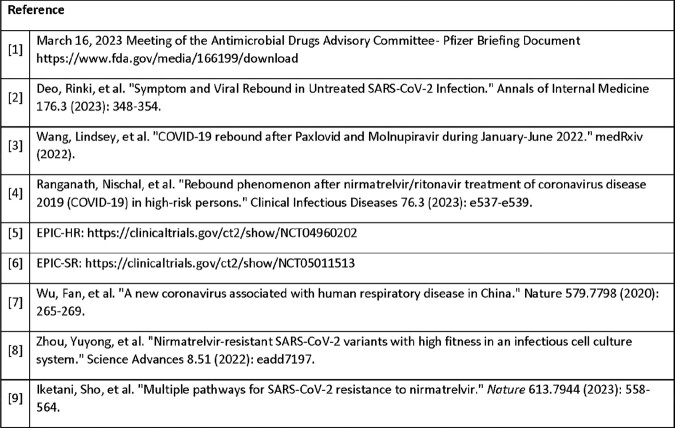

**Methods:**

Next generation sequencing analysis was performed from nasal swabs (D1, D3, D5, D10 and D14) with a viral RNA level ≥3.0 log_10_ copies/mL. The amino acid (AA) sequence was compared to Wuhan-Hu-1 [7] and M^pro^ substitutions were called if AAFREQ ≥ 10%. Emergent substitutions (ES) were called if observed post-baseline only and called treatment emergent substitutions (TES) if the ES was ≥ 2.5-fold more frequent, and had ≥3 additional occurrences, in nirm/r than placebo (PBO). Viral RNA load rebound (VLR) was defined as VL increase at D10 or D14 by ≥ 0.5 log_10_ copies/mL from D5 and resulting in a VL ≥ 3.0 log_10_ copies/mL.

**Results:**

In EPIC-HR/SR, the primary SARS-CoV-2 variant was Delta (91%) followed by Omicron (7.4%). The percentage of patients with M^pro^ ES did not differ between treatment arms. Among those with sequence data available (n=898 nirm/r, n=938 PBO), nirm/r M^pro^ TES were observed in: T98I/del(n=3), E166V (n=3), and W207L/del (n=3) and within the M^pro^ cleavage sites: A5328S/V(n=5) and S6799A/P/Y (n=4). None of the TES were associated with progression to severe COVID-19. VLR was observed in (6.1% nirm/r, 4.4% PBO) with M^pro^ TES observed in few VLR patients (3.4% in nirm/r and 0% in PBO). Among the M^pro^ TES identified, E166V is an *in vitro* resistance mutation [8,9]. Figure 1 shows the VL trajectories of the 3 patients with E166V, one patient experienced VLR at D10, all effectively controlled the virus by D14 and did not experience severe COVID-19.

**Figure:**
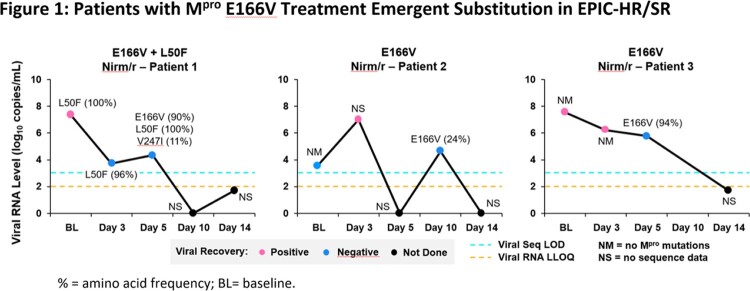

**Conclusion:**

In EPIC-HR/SR, an *in vitro* resistance substitution emerged in few patients, but was not associated severe COVID-19. Additionally, these data show VLR is not a result of treatment resistance to nirm/r driven by TES or any M^pro^ substitutions.

**Disclosures:**

**Mary Lynn Baniecki, PhD**, Pfizer Inc: Employee|Pfizer Inc: Stocks/Bonds **Shunjie Guan, PhD in Statistics**, AbbVie: Stocks/Bonds|Pfizer: Stocks/Bonds **Zhenyu Wang, Ph.D.**, Alkermes: Stocks/Bonds|Moderna: Stocks/Bonds|Pfizer: Stocks/Bonds **Yan Chen, Ph. D**, Pfizer, Inc: Stocks/Bonds **Weihang Bao, PhD**, Pfizer, Inc.: Employee of Pfizer|Pfizer, Inc.: Stocks/Bonds **Wen He, PhD**, Pfizer Inc.: Stocks/Bonds **Elizabeth Dushin, PhD**, Pfizer Inc.: Stocks/Bonds **Craig Hyde, PhD**, Pfizer: Employed by Pfizer|Pfizer: Stocks/Bonds **Yuao Zhu, PhD**, Pfizer, Inc.: Stocks/Bonds **Rhonda D. Cardin, PhD**, Pfizer, Inc: Stocks/Bonds **Jennifer Hammond, PhD**, Pfizer: Employee|Pfizer: Stocks/Bonds **Sandeep Menon, PhD**, Pfizer: Stocks/Bonds **Charlotte Allerton, PhD**, Pfizer: Stocks/Bonds **Holly Soares, PhD**, Pfizer: Stocks/Bonds

